# Impact of CT reconstruction algorithm on auto‐segmentation performance

**DOI:** 10.1002/acm2.12710

**Published:** 2019-09-20

**Authors:** Claudia Miller, Daniel Mittelstaedt, Noel Black, Paul Klahr, Siamak Nejad‐Davarani, Heinrich Schulz, Liran Goshen, Xiaoxia Han, Ahmed I Ghanem, Eric D. Morris, Carri Glide‐Hurst

**Affiliations:** ^1^ Department of Radiation Oncology Henry Ford Cancer Institute Detroit MI USA; ^2^ Wayne State University Detroit MI USA; ^3^ Department of CT Imaging Physics Philips Healthcare Cleveland OH USA; ^4^ Philips GmbH Innovative Technologies Hamburg Germany; ^5^ Department of Public Health Sciences Henry Ford Health System Detroit MI USA; ^6^ Clinical Oncology Department Alexandria University Alexandria Egypt

**Keywords:** auto‐segmentation, computed tomography, model‐based iterative reconstruction, reconstruction

## Abstract

Model‐based iterative reconstruction (MBIR) reduces CT imaging dose while maintaining image quality. However, MBIR reduces noise while preserving edges which may impact intensity‐based tasks such as auto‐segmentation. This work evaluates the sensitivity of an auto‐contouring prostate atlas across multiple MBIR reconstruction protocols and benchmarks the results against filtered back projection (FBP). Images were created from raw projection data for 11 prostate cancer cases using FBP and nine different MBIR reconstructions (3 protocols/3 noise reduction levels) yielding 10 reconstructions/patient. Five bony structures, bladder, rectum, prostate, and seminal vesicles (SVs) were segmented using an auto‐segmentation pipeline that renders 3D binary masks for analysis. Performance was evaluated for volume percent difference (VPD) and Dice similarity coefficient (DSC), using FBP as the gold standard. Nonparametric Friedman tests plus post hoc all pairwise comparisons were employed to test for significant differences (*P* < 0.05) for soft tissue organs and protocol/level combinations. A physician performed qualitative grading of 396 MBIR contours across the prostate, bladder, SVs, and rectum in comparison to FBP using a six‐point scale. MBIR contours agreed with FBP for bony anatomy (DSC ≥ 0.98), bladder (DSC ≥ 0.94, VPD < 8.5%), and prostate (DSC = 0.94 ± 0.03, VPD = 4.50 ± 4.77% (range: 0.07–26.39%). Increased variability was observed for rectum (VPD = 7.50 ± 7.56% and DSC = 0.90 ± 0.08) and SVs (VPD and DSC of 8.23 ± 9.86% range (0.00–35.80%) and 0.87 ± 0.11, respectively). Over the all protocol/level comparisons, a significant difference was observed for the prostate VPD between BSPL1 and BSTL2 (adjusted *P*‐value = 0.039). Nevertheless, 300 of 396 (75.8%) of the four soft tissue structures using MBIR were graded as equivalent or better than FBP, suggesting that MBIR offered potential improvements in auto‐segmentation performance when compared to FBP. Future work may involve tuning organ‐specific MBIR parameters to further improve auto‐segmentation performance. Running title: Impact of CT Reconstruction Algorithm on Auto‐segmentation Performance.

## 1 INTRODUCTION

One of the largest sources of uncertainty in radiation therapy planning (RTP) is the delineation of the target and organs at risk (OARs) using computed tomography (CT) datasets.^1^ Aside from the uncertainty introduced in the delineation process, manual delineation of OARs is time‐consuming.^2^ Thus, efforts to implement auto‐segmentation routines are advantageous and have shown promise for several disease sites, most commonly for head and neck, and prostate cancers.^3^, ^4^, ^5^ Current approaches to automated segmentation are commonly atlas‐based or a combination of atlas‐ and model‐based techniques.^3^ In the pelvis, auto‐segmentation has yielded good overall performance (~3 mm distance to mean surface in prostate segmentation from mean expert delineations).^6^, ^7^ Atlas‐based segmentation algorithms have appeared promising for the segmentation of bladder, rectum, and prostate (Dice similarity coefficient (DSC) > 0.70 with respect to radiation oncologist ground truth delineations) for prostate cancer treatment planning.^8^ Another auto‐segmentation toolkit, Smart Probabilistic Image Contouring Engine (SPICE), has been applied to CT scans in various disease sites and has demonstrated promise for clinical utility.^9^, ^10^

Current efforts are being made to move toward lower dose CT scanning, although image noise can be a rate‐limiting step due to the use of filtered back projection (FBP) for image reconstruction in CT simulation (CT‐SIM) datasets.^11^ One potential way to overcome image noise is to employ advanced reconstruction algorithms to maintain the same image quality while increasing the contrast to noise ratio.^12^, ^13^ Advanced CT reconstruction methods such as hybrid iterative reconstructions (HIR), model‐based iterative reconstruction (MBIR), and adaptive statistical iterative reconstruction (ASIR) have been integrated into clinical diagnostic CT scanners,^14^, ^15^ whereas their application in radiation oncology has been limited to date. In a study conducted by Price *et al*., HIR was found to maintain image quality with dose reduction protocols of up to ~ 70% when compared to FBP for CT‐SIM datasets in the female pelvis.^16^ While dose reduction is possible and MBIR has shown improvements in diagnosis and image quality,^17^ advanced iterative reconstruction algorithms have been shown to change the overall texture of datasets compared to standard FBP,^13^, ^18^, ^19^ which may lead to differences in intensity‐dependent automatic segmentation routines.

This study aims to evaluate the sensitivity of an auto‐segmentation algorithm to MBIR CT reconstructions for prostate cancer treatment planning and compare the results to the standard of care in CT‐SIM (FBP). With a better understanding of the impact of reconstruction methods on auto‐segmentation performance, the utility and potential application of advanced reconstruction algorithms may be integrated into the RTP workflow.

## 2 MATERIALS AND METHODS

### 2.1 CT‐Simulation and patient cohort

Eleven prostate cancer patients underwent CT simulation to generate a patient model for external beam treatment planning using a Brilliance Big Bore (Philips Health Care, Cleveland, OH) scanner positioned supine with the following parameters: 120–140 kVp, 500 mAs, 512 × 512 in‐plane image dimensions, 1.28 × 1.28 mm^2^ in‐plane spatial resolution, and 3 mm slice thickness. Patients were immobilized using bands placed around the feet, a ring to hold on their chest, and a shaped foam pad for leg immobilization. Raw sinogram data were exported from the clinical scanner and de‐identified for further processing.

### 2.2 Model‐based image reconstructions

Raw sinogram data were retrospectively reconstructed using FBP and MBIR algorithms with varied parameters using research reconstruction software (IMR, Philips Medical Systems, Cleveland, OH). The Philips Big Bore scanner specifies three user MBIR reconstruction protocols: Body Soft Tissue (BST), Body Routine (BR), and Body Sharp Plus (BSP). Each protocol is distinguished primarily by the reconstruction filter. Choice of filter reflects a tradeoff between noise and contrast resolution, BSP having the highest noise and resolution.^20^, ^21^ The MBIR reconstruction optimization equation is defined as:

$$F\left(x\right)=D\left(x\right)+\beta \cdot R\left(x\right)$$

where the function *F(x)* is composed of a data fit term *D(x)* and a noise reducing (but edge preserving) regularization term *R(x)* with strength controlled by the factor β. For FBP, image noise is known to scale with well‐defined ratios based on slice thickness, patient size, and mAs. While noise reductions arising from the MBIR reconstruction levels (L1‐L3) depend on acquisition parameters including slice thickness, mAs, and patient size, they do not scale in proportion to the FBP noise.^22^ Nevertheless, as the underlying raw data acquisition parameters were fixed in this study, higher level reconstructions (i.e., L3) were expected to have the largest reduction in noise, particularly for low dose protocols (e.g., ~60% noise reduction between L1 and L3 for an abdomen CT acquired at 300 mAs, 1 mm, Body Routine filter acquired with an Ingenuity CT scanner^22^).

### 2.3 Organ auto‐segmentation

For each reconstructed CT image, auto‐segmentation was performed using a research prototype version of SPICE software (Philips, Cleveland, OH). The prostate cancer cases produced auto‐segmentations for nine OARs (four soft tissue (prostate, bladder, rectum, and seminal vesicles) and five bony structures (left and right sides of the pelvis and femur, as well as the sacrum). The segmentation software pipeline includes three main steps: (a) global positioning, (b) organ‐specific positioning, and (c) structure refinement, as described by Bzdusek *et al*.^23^ Briefly, the first step rotates, translates, and performs an isotropic scaling registration of a tissue probability atlas to a tissue classified target image. In the second step, the organs are positioned while using both the tissue classified image and the organ‐specific probability maps. Lastly, in the third step, model‐based segmentation is used to bring the surface mesh triangles to trained image features.^23^

Comparisons were performed to investigate the differences in each organ segmentation (e.g., prostate, rectum) based upon the MBIR protocol and level. The auto‐segmentations were compared for the different MBIR reconstructions of the identical raw dataset, as well as between patients to further understand the impact of the CT reconstruction on auto‐segmentation of organs, and patient‐specific differences.

### 2.4 Physician qualitative contour grading

Eleven prostate cancer patient datasets (396 generated contours (99 prostate, rectum, seminal vesicles, and bladder)) were qualitatively evaluated by a physician instructed to grade the generated MBIR contours at each protocol/level in comparison to those generated on the reference dataset (i.e., FBP) using a six‐point scale (1: Better than reference, 2: Slightly better than reference, 3: Equivalent to reference, 4: Slightly worse than reference, 5: Worse than reference, 6: Clinically unacceptable).^16^, ^18^ A score of six was also assigned to contours that incorrectly segmented the organ regardless of agreement to FBP. During the contour review, the physician was blinded to reconstruction protocol/level. After review of the grading results, any contour that was deemed clinically unacceptable (score of 6) was removed from the patient cohort for any further statistical evaluation to ensure that only clinically acceptable data fit for review were maintained within the cohort.

### 2.5 Analyses and quantitative comparisons

The auto‐segmentations from each of the nine different MBIR reconstructions and FBP were quantitatively evaluated using volume percent difference (VPD) and DSC. For each prostate cancer case, the auto‐segmentations were analyzed using an in‐house MATLAB program (Mathworks, Natick, MA) to measure the volume of the OARs. VPD was calculated with the FBP being the reference as defined:

$$VPD=ABS\left(\frac{MBIR_{\mathrm{volume}}-FBP_{\mathrm{volume}}}{FBP_{\mathrm{volume}}}\right)\times 100$$

where $MBIR_{\mathrm{volume}}$ and $FBP_{\mathrm{volume}}$ are the measured auto‐segmented volumes of an organ using the MBIR and FBP reconstructions, respectively.

The resulting segmentations were also quantitatively compared using the DSC to assess the regional overlap between the organs according to:

$$DSC=2\times \frac{\left|A\cap B\right|}{\left|A\right|+\left|B\right|}$$

In this equation, A and B are the volumes of the reference (i.e., FBP) and MBIR auto‐segmented organ respectively, and A∩B is defined as the volume of the intersection of the auto‐segmented organ from the two reconstruction methods. DSC equal to zero describes no overlap and DSC equal to 1 demonstrates complete agreement of the auto‐segmentations. Finally, center of mass (COM) comparisons were made between the auto‐segmented organs from FBP and MBIR reconstructions to elucidate potential location differences across each major axes (X‐ (right‐left), Y‐(anterior‐posterior), and Z‐(superior‐inferior)).

### 2.6 Analyses for statistical comparisons

To determine differences among the nine MBIR protocols, since the resulting index values were not normally distributed, a nonparametric Friedman test plus post hoc for all pairwise comparisons was employed.^24^ To test for differences among protocols, three pairwise comparisons were conducted over all soft tissue organs. To test for significant differences among protocol/level combinations, 36 pairwise comparisons were evaluated. Data were presented as mean ± SD (standard deviation) and interquartile range (IQR). Adjusted *P*‐values using Bonferroni corrections for multiple tests were used and *P*‐values less than 0.05 (two‐tailed) were regarded as statistically significant. All statistical analysis was performed using SPSS version 25.0 (SPSS, Chicago, IL, USA).

## 3 RESULTS

**Figure 1:**
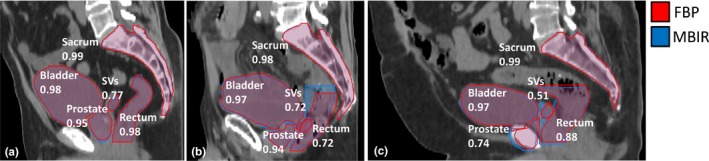
Comparison between auto‐segmentation results for three different patient cases (Body Sharp Plus protocol at L1). Each image has both the filtered back projection (FBP) and model‐based iterative reconstruction (MBIR) contours shown with (a) an average patient result, (b) abnormal case with an abnormally full rectum, adversely impacting the auto‐segmentation performance for MBIR, and (c) patient with a high body mass index where the seminal vesicles (SVs) had worse agreement and the pubic symphysis was incorrectly identified as the prostate for both reconstructions. Numerical values labeling each organ represent the Dice similarity coefficient (DSC) value for comparison of MBIR to FBP.

**Figure 2:**
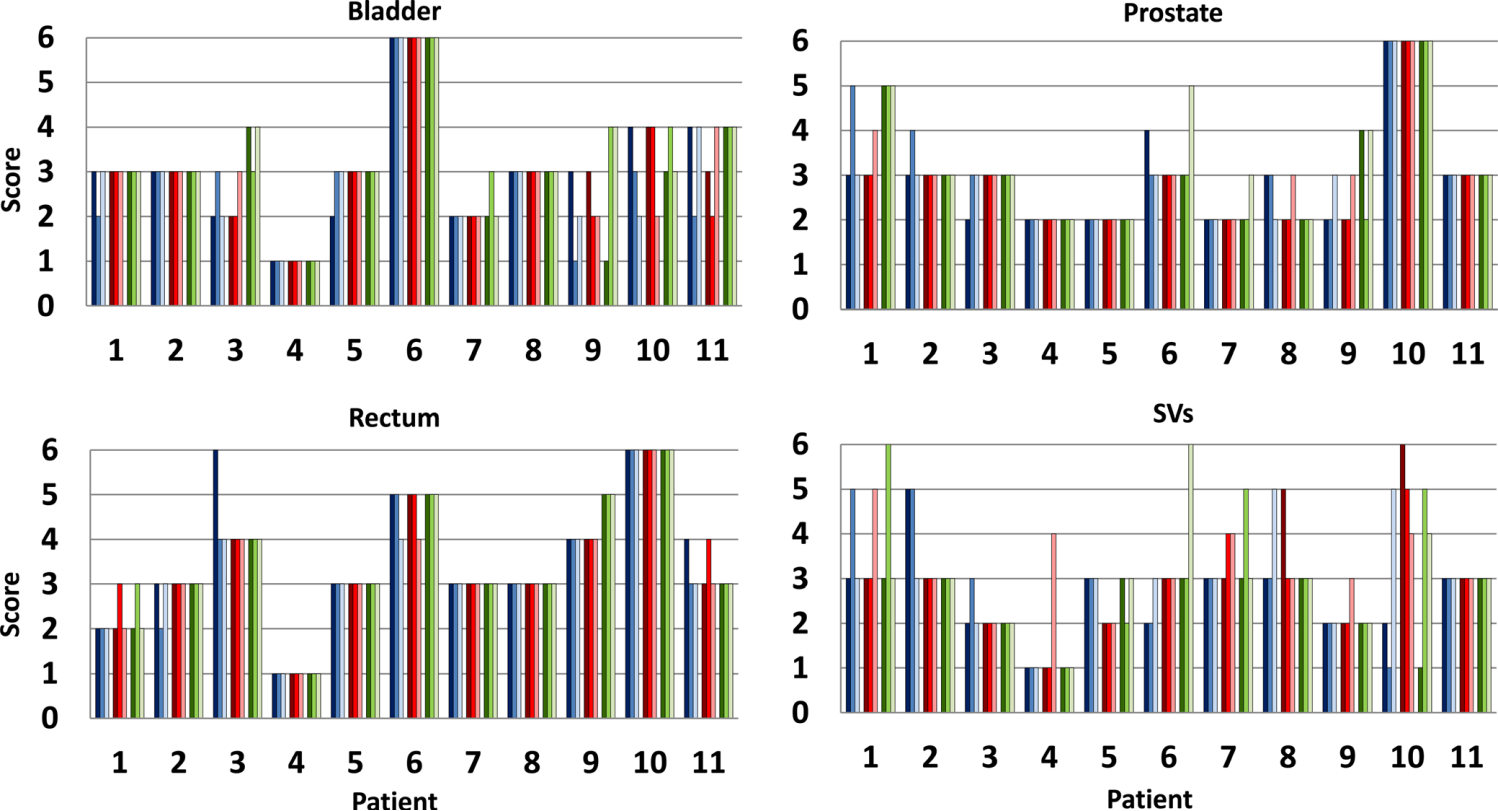
Physician grading scores using a 6‐point scale (1: Better than reference, 2: Slightly better than reference, 3: Equivalent to reference, 4: Slightly worse than reference, 5: Worse than reference, 6: Clinically unacceptable) for bladder, prostate, rectum, and seminal vesicles (SVs). This was done for all 11 patients for each of the nine protocol/level combinations which yielded 396 contours for evaluation. Protocol and level combinations defined in text.

All prostate cancer patient CT reconstructions were able to be segmented by SPICE; however, 27 organ contours were deemed “clinically unacceptable” by physician grading and were thus removed from further quantitative evaluation. Of these 27 contours, 23 were graded as clinically unacceptable for both MBIR and FBP. One such patient had a high body mass index which caused the pubic symphysis to be incorrectly identified as the prostate for both FBP and MBIR reconstructions reconstructions [Fig. 1(c) and Fig. 2, Patient 10]. Another case that was graded clinically unacceptable is shown in [Fig. 1(b)] where the patient had an abnormally full rectum that adversely impacted auto‐segmentation performance as also indicated in Fig. 2, Patient 6. A total of 369 out of 396 (93%) soft tissue contours were clinically useable (score of 1‐5) and included in all subsequent quantitative analysis. Of the 4 scores that were scored as adversely impacted by MBIR as compared to FBP, 3 were seminal vesicles and 1 was a rectum case. Nevertheless, Fig. 2 highlights that 75.8% of MBIR auto‐segmentations were scored equivalent or better than FBP across the patient cohort. Notably, 30.6% of the MBIR segmentations were graded as better or slightly better than FBP, particularly for the prostate (38/99), SVs (33/99), and bladder (32/99).

The highly attenuating and high contrast structures (i.e., bony anatomy) yielded similar auto‐segmentation results between the FBP and MBIR reconstruction parameters (DSC > 0.98). Generally speaking, the bladder was also insensitive to reconstruction protocol, yielding the least amount of changes for the soft tissues between the FBP and MBIR reconstructions (VPD = 2.15 ± 2.42%, range: 0.01–8.47%). A high degree of overlap was observed for the bladder across all reconstructions (DSC = 0.97 ± 0.01, range: 0.94–0.99). One subject had a maximum COM difference of 3.06 mm for this soft tissue reconstruction.

**Figure 3:**
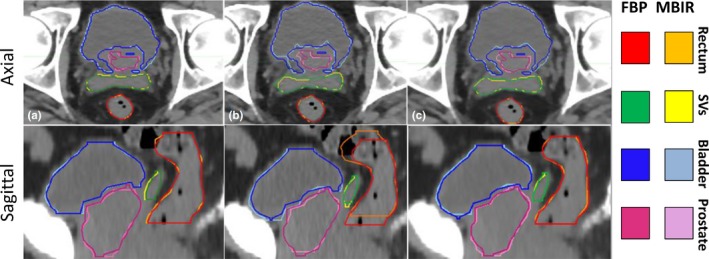
Model‐based iterative reconstructions and accompanied auto‐segmentations for the (a) Body Routine, (b) Body Sharp Plus, and (c) Body Soft Tissue protocols at L1 as compared to filtered back projection (FBP) segmentations.

Table 2 summarizes the MBIR auto‐segmentation results (VPD, DSC, and COM) for the soft tissue organs by protocol as compared to FBP for the 11 patients. Figure 3 highlights typical auto‐segmentation results for FBP and MBIR reconstructions across the three different protocols evaluated. Over all 11 patients, the rectum had an average VPD of 7.50 ± 7.56% (range: 0.11–42.80%). The average DSC values were 0.90 ± 0.08 (range: 0.66–0.98). The rectum yielded the largest COM shifts between MBIR reconstructions and FBP (average shift of 4.5 mm) largely due to deviations in the superior/inferior extent as shown in the sagittal images of Fig. 3(b) where discrepancies in the superior and inferior extents were observed between the FBP and MBIR BSP protocol.

**Table 1:** The average of the volume percent difference (VPD) magnitude, Dice similarity coefficient (DSC), and distance between the model‐based iterative reconstruction (MBIR) and filtered back projection (FBP) center of mass (COM) all with standard deviations (SD) based on the MBIR and FBP reconstruction methods.

		ABS VPD (%)	DSC (A.U.)	COM (mm)
		MEAN +/‒ SD	MEAN +/‒ SD	MEAN +/‒ SD
Organ	Protocol	Range (min, max)	Range (min, max)	Range (min, max)
Bladder	BR	2.20 ± 2.62	0.98 ± 0.01	0.69 ± 0.76
		(0.01, 8.47)	(0.95, 0.99)	(0.11, 2.84)
	BSP	1.82 ± 2.21	0.98 ± 0.01	0.65 ± 0.75
		(0.04, 7.81)	(0.95, 0.99)	(0.05, 2.75)
	BST	2.44 ± 2.44	0.97 ± 0.01	0.74 ± 0.79
		(0.17, 8.04)	(0.94, 0.99)	(0.11, 3.06)
Prostate	BR	3.53 ± 3.40	0.94 ± 0.03	0.89 ± 0.97
		(0.07, 17.16)	(0.87, 0.97)	(0.07, 3.61)
	BSP	2.31 ± 2.12	0.94 ± 0.03	0.83 ± 0.84
		(0.07, 9.26)	(0.87, 0.97)	(0.08, 3.27)
	BST	7.65 ± 6.14	0.92 ± 0.04	1.24 ± 1.31
		(0.10, 26.39)	(0.77, 0.96)	(0.16, 4.95)
Rectum	BR	8.00 ± 9.31	0.91 ± 0.07	4.01 ± 3.94
		(0.11, 41.97)	(0.69, 0.98)	(0.14, 16.01)
	BSP	7.62 ± 7.84	0.90 ± 0.08	4.67 ± 3.59
		(0.41, 42.80)	(0.72, 0.98)	(0.11, 13.01)
	BST	6.91 ± 5.32	0.89 ± 0.08	4.87 ± 3.91
		(0.33, 21.30)	(0.66, 0.98)	(0.06, 14.51)
SVs	BR	9.41 ± 10.74	0.87 ± 0.10	2.22 ± 2.29
		(0.05, 34.69)	(0.61, 0.98)	(0.12, 7.54)
	BSP	7.19 ± 9.02	0.88 ± 0.09	1.97 ± 2.11
		(0.00, 32.82)	(0.63, 0.96)	(0.11, 7.98)
	BST	8.05 ± 9.90	0.86 ± 0.14	2.37 ± 2.85
		(0.16, 35.80)	(0.48, 0.97)	(0.15, 9.58)

For the prostate, the average magnitude of the VPD was 4.50 ± 4.77% (range: 0.07–26.39%). The prostate measured average DSC values to be 0.94 ± 0.03 (range: 0.77–0.97). There was a maximum COM difference of 4.95 mm between the FBP and MBIR. Auto‐segmentation results for the seminal vesicles yielded a mean VPD of 8.23 ± 9.86% (range: 0.00–35.80%) with an average DSC of 0.87 ± 0.11 (range: 0.48–0.98). When evaluating the impact of the three MBIR protocols (BR, BSP, BST) on soft tissue segmentation, there were no observed differences for rectum, bladder, and SVs when analyzing comparisons. The prostate VPD experienced significant differences between the BSPL1 and BSTL2 combinations (adjusted *P*‐value = 0.039).

**Figure 4:**
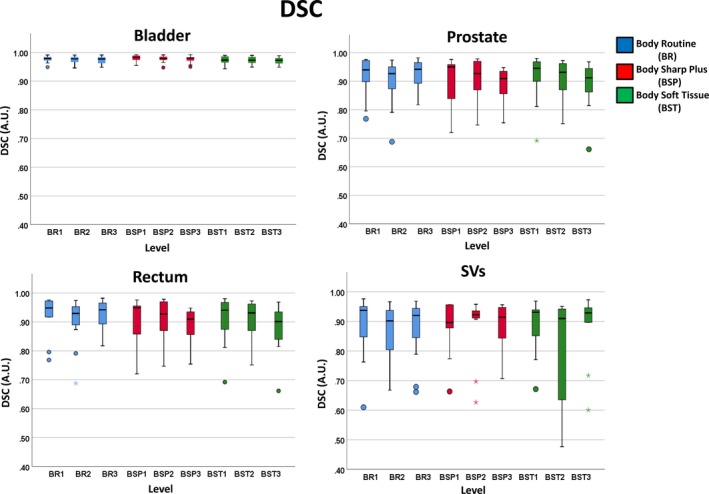
Dice similarity coefficient (DSC) boxplot comparison between the four soft tissue organs: bladder, prostate, rectum, and seminal vesicles (SVs), for each protocol type and level for 11 prostate cancer patients. Boxplots, thick line, and whiskers represent the interquartile range, median, and 5th and 95th percentiles, respectively. Data points displayed as a small circle represent a value >1.5 times the interquartile range (IQR) and the star represents a value >3×IQR.

**Figure 5:**
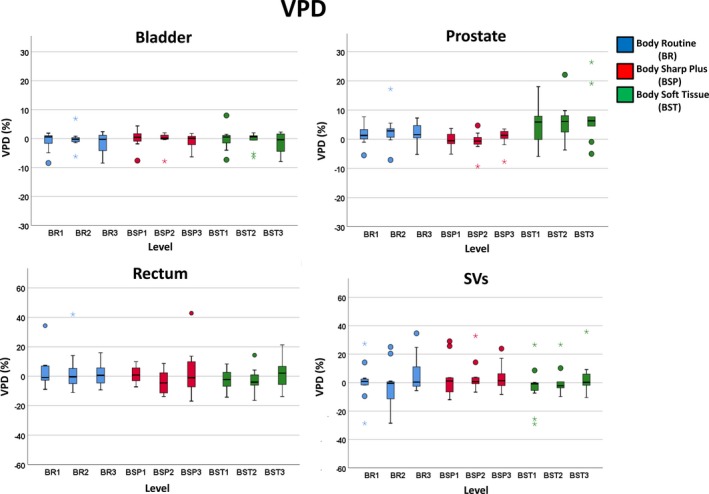
Volume percent difference (VPD) boxplot comparison between the four soft tissue organs: Bladder, prostate, rectum, and seminal vesicles (SVs), for each protocol type and level for 11 prostate cancer patients with the top row displaying different Y‐axis magnitude than the bottom row. Boxplots, thick line, and whiskers represent the interquartile range, median, and 5th and 95th percentiles, respectively. Data points displayed as a small circle represent a value >1.5 times the interquartile range (IQR) and the star represents a value >3×IQR.

These sensitivity results demonstrate differences between the FBP and MBIR CT reconstruction algorithms for auto‐segmentation, further described by Table 2 for the COM differences in the X‐ (right‐left), Y‐(anterior‐posterior), and Z‐(superior‐inferior) directions. There were minor differences noticed in the COM X‐, Y‐, and Z‐directions for the bladder (mean of 0.69 mm) and prostate (mean of 0.99 mm), however one outlier was observed for the prostate (COM deviation of up to −106.88 mm in the X). The rectum measured large changes in the superior‐inferior (Z‐direction) with COM differences ranging from −31.40 to 15.91 mm, suggesting challenges in identifying the upper and lower bounds of the organ. The SVs demonstrated large variation in the COM Z‐ and X‐directions with ranges of −4.23 to 51.85 mm and −7.20 to 8.01 mm, respectively. Figures 4 and 5 highlight boxplots indicating specific organ and protocol/level combination auto‐segmentation results for the cohort for both DSC and VPD.

## 4 DISCUSSION

This work sought to determine the impact of a model‐based iterative reconstruction algorithm on auto‐contouring performance for prostate cancer patients. Quantitative comparison between FBP and MBIR revealed that the soft tissue organs, such as the rectum, prostate, and SVs, experienced the greatest amount of segmentation variability. However, the high contrast bony structures were the least affected (average DSC > 0.98). A similar result was found by Delpon *et al.* that high attenuating structures, such as the pelvic bones, had a DSC of ~ 0.90 when compared to the physician delineations used in their study.^25^

Auto‐segmentation performance has also been shown to be impacted by organ size, as found by Kumarasiri *et al.* where soft tissue structures were classified by size for CT auto‐segmentation.^26^ In studies focusing on the pelvic region, similar results were found in that larger structures, such as the bladder, had smaller volume variations when compared to smaller structures similar to the prostate.^27^ This was also observed in our study where the bladder was more accurately contoured in comparison to the smaller structures (rectum, prostate, and SVs), as shown by the DSC results in Table 1 and the COM distance results shown in Table 2. Our results were comparable to work done by Isambert *et al.* in which the auto‐contouring software used was reliable for large structures, but still in need of additional delineation revisions by an expert for small, more complex structures.^2^ In addition to size, contrast also impacts auto‐segmentation performance, lending to more accurate bony segmentation observed in this study compared to all other organs.

**Table 2:** The average of the MBIR protocols for the magnitude of the distance between the MBIR and FBP COM with SD for the X‐ (right‐left), Y‐(anterior‐posterior), and Z‐(superior‐inferior) axes. The range includes the minimum and maximum distances of the measured values. All measurements are in millimeters (mm).

		COM X	COM Y	COM Z
		MEAN +/‒ SD	MEAN +/‒ SD	MEAN +/‒ SD
Organ	Protocol	Range (min, max)	Range (min, max)	Range (min, max)
Bladder	BR	0.04 ± 0.18	0.27 ± 0.89	0.22 ± 0.43
		(−0.33, 0.38)	(−0.40, 2.72)	(−0.75, 0.97)
	BSP	0.02 ± 0.17	0.28 ± 0.81	0.11 ± 0.46
		(−0.26, 0.40)	(−0.40, 2.66)	(−0.67, 1.59)
	BST	−0.01 ± 0.16	0.27 ± 0.89	0.24 ± 0.49
		(−0.41, 0.30)	(−0.62, 3.00)	(−0.58, 1.09)
Prostate	BR	−0.03 ± 0.73	0.08 ± 0.53	0.22 ± 0.87
		(−1.25, 3.27)	(−0.51, 1.65)	(−1.53, 2.22)
	BSP	−0.16 ± 0.43	0.06 ± 0.51	0.30 ± 0.92
		(−1.60, 0.41)	(−0.68, 1.56)	(−1.26, 2.74)
	BST	−3.20 ± 19.63	0.20 ± 0.80	−0.05 ± 2.05
		(−106.88, 4.22)	(−0.68, 3.78)	(−9.40, 2.65)
Rectum	BR	0.14 ± 0.65	−0.05 ± 0.81	1.48 ± 5.36
		(−0.64, 2.71)	(−1.77, 1.95)	(−7.27, 15.91)
	BSP	0.39 ± 1.66	0.27 ± 1.29	1.15 ± 5.43
		(−1.51, 6.65)	(−3.06, 3.59)	(−10.43, 12.65)
	BST	3.50 ± 18.18	1.66 ± 9.32	−0.44 ± 8.08
		(−0.62, 102.76)	(−2.87, 52.26)	(−31.40, 13.96)
SVs	BR	0.84 ± 2.32	0.12 ± 0.77	0.27 ± 1.88
		(−5.83, 6.84)	(−0.81, 2.68)	(−3.84, 5.19)
	BSP	0.63 ± 1.96	0.14 ± 0.99	0.36 ± 1.38
		(−3.33, 7.56)	(−0.68, 4.11)	(−1.35, 5.02)
	BST	0.52 ± 2.68	1.03 ± 5.47	2.06 ± 9.45
		(−7.20, 8.01)	(−3.12, 29.35)	(−4.23, 51.85)

BSP, body sharp plus; BST, body soft tissue; COM, center of mass; FBP, filtered back projection; MBIR; model‐based iterative reconstruction; SD, standard deviations; SVs, seminal vesicles.

Our statistical results showed us that between the protocol/level combinations there were significant differences for reconstructions in both the bladder and the prostate. This suggests that there may be a specific protocol that is optimal for prostate auto‐segmentation tasks and that using iterative reconstruction techniques may be advantageous to these RT tasks as well. While limited segmentation data are available for direct comparison, several groups have evaluated the impact of model‐based iterative reconstruction on image quality tasks. For example, FBP has been found to have inferior image quality as compared to both MBIR and hybrid iterative reconstruction (HIR) and that MBIR yielded superior image quality to HIR for the cross‐sectional view.^15^ A study conducted by Hèrin et al. concluded that MBIR reconstructions of reduced‐dose CT and FBP reconstruction of standard‐dose CT both obtained the same low contrast detectability on a phantom.^28^ This suggests that model‐based iterative reconstruction methods have the potential to perform better than FBP in terms of overall image quality and thus may impact Radiation Oncology‐related tasks. A limitation of our work is the sample size (11 evaluable patients). However, 11 patients were reconstructed with nine protocol/level combinations with the four soft tissue structures evaluated yielding a total of 396 data points for review. Conducting this evaluation on a larger sample size may be advantageous to determine any further statistical significance in our findings.

Our work on the analysis of nine MBIR reconstruction protocol/level combinations differs from previously reported work by examining the application to the RTP‐specific task of auto‐contouring. MBIR has been previously shown to improve image quality by reducing noise and enabling the acquisition of lower dose scans that can substantially reduce imaging dose when compared to FBP.^29^, ^30^ The ability to reduce imaging radiation dose following as low as reasonably acceptable (ALARA) and “imaging gently” procedures will reduce the risk of overexposure to the patient. Reducing radiation exposure can still allow for appropriate OAR segmentations by improving reconstruction algorithms, such as MBIR.

In the cohort evaluated, while all patient CT reconstructions were able to be successfully segmented by SPICE, some erroneous segmentation results were encountered. There were 27 (6.82%) soft tissue contours that received a score of “Clinically Unacceptable” although only four of them were attributed to MBIR while the remaining 23 were due to abnormal patient anatomy. This suggests that in cases where the atlas cannot adequately perform auto‐segmentation, the reconstruction protocol will not offer improvement to the overall auto‐segmentation performance. A similar result was found in a study conducted by McBain *et al.* in that irregularly shaped anatomy was not properly contoured by an automatic contouring system.^31^ Nevertheless, out of the 396 generated contours for the four soft tissue structures using MBIR, 300 (75.8%) of them were graded as equivalent to or better than FBP. Notably, ~38% of prostate and ~ 32–33% of bladder and SV contours segmented on MBIR data scored qualitatively better/slightly better than the FBP corresponding contours, suggesting that MBIR offered improvement in auto‐segmentation performance as compared to FBP. The ability to properly segment the OAR with automatic segmentation increases the efficiency of RTP by allowing the physician to spend less time manually delineating each organ needed for dose calculations which is described by La Macchia et al. in which the automatic contouring workflow was shown to be significantly shorter than the manual contouring process.^9^

An additional limitation of this work is that all MBIR segmentation results were reported in reference to FBP. Although FBP is considered the gold standard for radiation oncology delineations,^32^ FBP may be limited by sensitivity to noise, motion, metal, and streak artifacts.^33^ Nevertheless, this work incorporated qualitative scoring by a physician to assess differences between FBP and MBIR results. Work to integrate advanced reconstruction algorithms into CT‐SIM platforms is ongoing.^16^ Future work can build on our qualitative grading by incorporating physician‐based ground truth delineations to find the most promising MBIR algorithm combinations for different contouring endpoints. Although our study did not use physician delineations, the quantitative differences found between FBP and MBIR demonstrated for both VPD and DSC show that the advanced reconstruction algorithms are providing images with different characteristics than the FBP reconstruction and thus have an impact on auto‐segmentation for lower contrast organs such as the prostate and SVs. Additionally, more research on MBIR reconstruction is needed to further investigate how it can be used or improved to be implemented in the treatment or location detection of a specific organ. As MBIR makes its way into Radiation Oncology CT‐SIM platforms to enable reductions in imaging dose, the impact on auto‐segmentation task performance will be of increasing importance for clinical efficiency. This work revealed that auto‐segmentation performance on MBIR images was comparable or better than FBP for 75% of the generated soft tissue contours, although more complex structures, such as the SVs may still require manual edits.

## 5 CONCLUSION

Automatic segmentation for MBIR on high contrast structures was successful and offered improved segmentation quality for 30‐40% of the bladder, prostate, and SV contours as compared to FBP. Although manual modifications may still be necessary, when coupling MBIR with auto‐segmentation, both imaging dose and treatment planning time are reduced. Future work may involve selecting organ‐specific MBIR parameters to improve auto‐segmentation performance.

## CONFLICT OF INTEREST

The submitting institution holds research agreements with Philips Healthcare. Noel Black, Paul Klahr, Heinrich Schulz, and Liran Goshen are clinical scientist employees of Philips Healthcare.
